# Same-day Discharge after Subcutaneous Implantable Cardioverter-defibrillator Implantation is Safe and Cost-effective

**DOI:** 10.19102/icrm.2020.110602

**Published:** 2020-06-15

**Authors:** Jill Swinning, Kelli Fox, Sreedhar Billakanty, Shannon Brown, Nagesh Chopra, Eugene Fu, Jennifer James, Gregory Kidwell, James Kleman, Victoria Murnane, Steven Nelson, Allan Nichols, S. Kay Nichols, Andrea Robinson, Anish Amin

**Affiliations:** ^1^OhioHealth Heart and Vascular Physicians, Riverside Methodist Hospital, Columbus, OH, USA

**Keywords:** Cardiac implantable electronic device, same-day discharge, subcutaneous implantable cardioverter-defibrillator, sudden cardiac death

## Abstract

The feasibility and safety of same-day discharge after transvenous implantable cardioverter-defibrillator implantation is well-established. However, subcutaneous ICDs (S-ICDs) are now increasingly being implanted, and the feasibility, safety, and potential cost savings associated with same-day discharge after S-ICD placement has not been widely investigated. In a small cohort of patients (n = 24) who underwent S-ICD implantation at our institution, 54% were successfully discharged on the same day as their implant procedure. Procedure-related complications were not apparent in this sampling and the reduction in health care costs was high, suggesting this protocol has immense benefit in today’s health care environment. As such, same-day discharge of S-ICD patients is appropriate to consider and should receive further attention.

## Introduction

Implantable cardioverter-defibrillators (ICDs) have been demonstrated to reduce the incidence of sudden cardiac death in select patients. The feasibility and safety of same-day discharge after transvenous ICD systems is well-established; same-day discharge after transvenous ICD implantation improves patient satisfaction, increases bed availability, and facilitates overall hospital cost savings without adversely affecting readmission rates.^1,2^ Subcutaneous ICDs (S-ICDs) are now increasingly being implanted as an alternative to transvenous ICDs as they are considered safe and effective in terminating lethal arrhythmias and they have comparable complication rates.^3,4^ In the adult population, it is not standard of practice to use general anesthesia during transvenous device implants. Historically, however, S-ICD patients have not been discharged on the day of implantation secondary to the use of general anesthesia to facilitate implantation. However, it may be reasonable to consider same-day discharge in select patients.

To our knowledge, the feasibility, safety, and potential cost savings associated with same-day discharge after S-ICD placement have not been investigated. Therefore, this study sought to evaluate the safety, feasibility, and cost reductions associated with same-day discharge after S-ICD implantation for primary prevention in comparison with those among patients observed overnight after implantation.

## Methods

We prospectively analyzed 24 consecutive patients who presented to our institution to solely undergo S-ICD implantation for a primary prevention indication over an eight-month period. This study was reviewed by the Office of Human Subjects Protection of our research institute and was deemed to be a quality improvement study and not a human research study so therefore did not require institutional review board approval. Patients were excluded if they were scheduled for a lead or device revision, lived more than 30 miles from an emergency department, did not have a driver or responsible party to stay with them overnight, developed a postprocedure complication while in the hospital, or the procedure was completed after 1:00 PM.

A protocol was developed and implemented for same-day discharge of S-ICD patients. The implanting physician, rounding nurse practitioner, electrophysiology laboratory staff, and device clinic staff were all assigned specific responsibilities to ensure smooth same-day discharge and coordination for follow-up. The nurse practitioner was responsible for evaluation of the patient prior to discharge, reviewing the same-day device interrogation and chest X-ray, and providing device education including activity restrictions and follow-up details to the patient. The patient also received a follow-up telephone call from the nurse practitioner the next day. The patient was scheduled to undergo an incision check one week after at the practice device clinic and an in-clinic device interrogation three months after device implantation, respectively.

The 24 patients were 17% female and 83% male, with ages ranging from 23 to 82 years (average age: 62.1 years). As stated, all devices were implanted for a primary prevention indication. Fifteen (62.5%) patients had a diagnosis of nonischemic cardiomyopathy (NICM) and nine (37.5%) had that of ischemic cardiomyopathy (ICM). The average ejection fraction for these patients was 27.8% (range: 15%–35%). One patient had a prior ICD, extracted in a previous setting.

The study participants were divided into two groups: group A consisted of patients successfully discharged on the same day (n = 13 patients) and group B consisted of patients observed overnight after implantation (n = 11 patients). Same-day discharge was defined as discharge home prior to 9:00 PM on the same calendar day as implantation.

For patients in both groups, electronic medical records were reviewed three months postimplantation. Outpatient and inpatient records during this time frame were reviewed to identify any complications potentially related to S-ICD implantation, including as surgical site bleeding, hematoma, lead dislodgement, infection, or cardiac injury.

## Results

Of the 24 patients included in this study, 54% were successfully discharged on the same day. Patients who were not discharged on the same day experienced excessive postprocedural pain and/or the patient/implanting physician preference mandated an overnight stay. No postprocedural complications were reported in the initial three-month period postimplantation for patients in either group. Of note, two patients in group A were readmitted within 30 days of discharge (one for an acute heart failure exacerbation at 20 days postimplantation and one for symptoms of fatigue at 15 days postimplantation). Neither of these readmittances were thought to be directly related to device implantation or would have necessarily been avoided had these patients stayed overnight after their procedure. Device interrogation or reprogramming prior to the initial three-month interrogation was not required in either group.

The demographics of the two groups were very similar. In group A, 53.8% of patients had NICM and 46.2% had ICM. In group B, 72.7% patients had NICM and 27.2% had ICM. Age, gender, and average ejection fraction were not a factor in predicting which patients were discharged on the day of implantation. The average ages in groups A and B were 60 years and 64.3 years, respectively, while the average ejection fractions were 27.4% and 25.4% **(Table 1)**.

**Figure 1:**
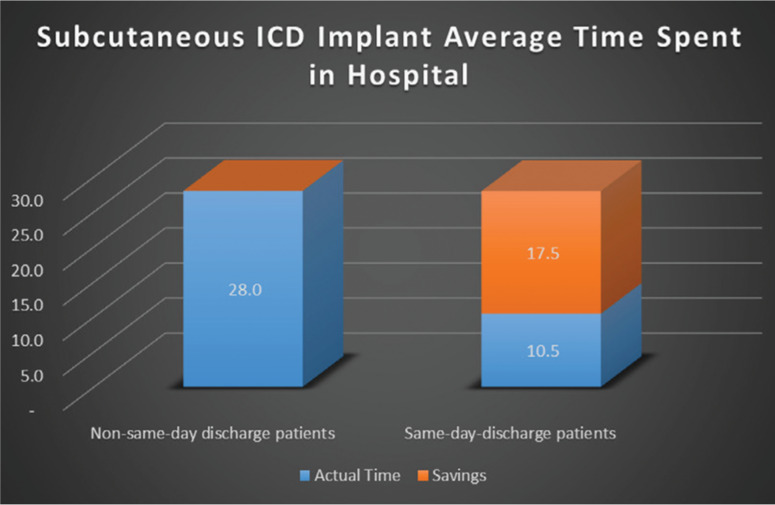
Comparison of the average time spent in the hospital between patients who stayed overnight and those who were discharged on the same day as device implantation.

**Figure 2:**
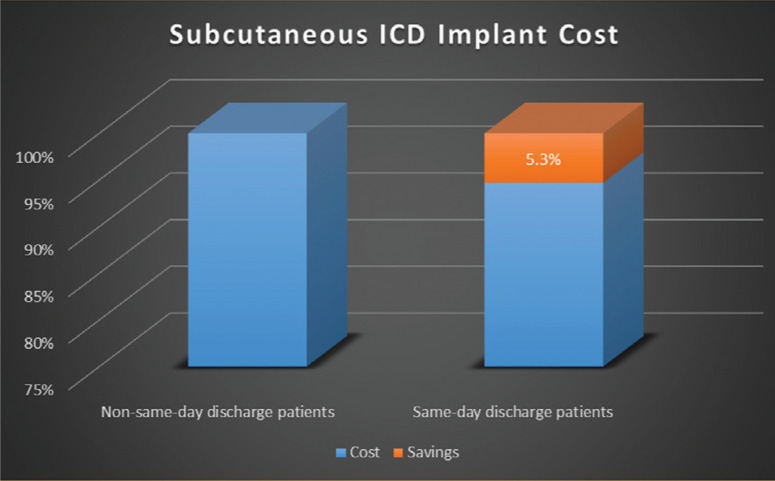
Comparison of the costs between patients who stayed overnight and those who were discharged on the same day as device implantation.

Separately, the average time spent in the hospital per patient following S-ICD implantation demonstrated a time savings of 63% when patients were discharged on the same day **(Figure 1)**. This improved hospital bed access by 17.5 hours per patient. The overall hospital savings achieved by discharging patients on the same day were calculated by comparing hospital costs and hospital charges. This calculation demonstrated a modest reduction of 5.3% in the cost of care per patient with discharge on the same day, or an average of $1,664.48 per patient **(Figure 2)**. A reduction in nursing costs largely accounted for these savings.

## Discussion

Current procedural billing and coding protocols for ICD implants classifies S-ICD implantation as an outpatient procedure, and, therefore, overnight resource utilization cannot be charged for (eg, nutrition services, respiratory therapy to set up a continuous positive-airway pressure machine). Consequently, all ICD implants are billed and coded the same regardless of the length of stay even though resource utilization is significantly less when a patient is discharged on the same day.

In an environment of increasingly strained health-care resources, it is paramount to recognize best practices that improve efficiency and economics. Implementing structured postprocedure protocols can reduce unnecessary hospitalizations. The use of telephone and telemedicine monitoring can further provide virtual follow-up to ensure patients’ postprocedural recovery is progressing as anticipated and no lingering questions remain. A potentially novel role for a postprocedure navigator may allow for expansion of same-day protocols and could improve the overall patient experience and care.

## Conclusion

It is appropriate to consider same-day discharge in select patients who are determined as eligible for such per institution discretion after undergoing S-ICD implantation for a primary prevention indication. In a small cohort of patients undergoing S-ICD implantation over an eight-month period, same-day discharge was safe and feasible. Procedure-related complications were not apparent, and a reduction in costs associated with health care may be realized as a result.

## Figures and Tables

**Table 1:** Patient Characteristics of Same-day Discharge (Group A) and Overnight Stay (Group B) Groups.

	All Patients (n = 24)	Group A (n = 13)	Group B (n = 11)
Male, %	83.3	85	82
Female, %	16.6	15	18
Average age, years	62.1	60	64.3
Ischemic cardiomyopathy, %	37.5	46.2	27.2
Nonischemic cardiomyopathy, %	62.5	53.8	72.7
Average ejection fraction, %	27.8	27.4	25.4
